# Effects of Motor Rehabilitation on Balance and Functional Activities in Elderly Patients with Peripheral Neuropathy and Recurrent Falls

**DOI:** 10.3390/life13041059

**Published:** 2023-04-20

**Authors:** Bernardo Gialanella, Laura Comini, Paola Prometti, Fabio Vanoglio, Raffaele Santoro

**Affiliations:** 1 Istituti Clinici Scientifici Maugeri IRCCS, Neurorehabilitation of the Institute of Lumezzane, 25065 Lumezzane, Italy; gialanellab@gmail.com (B.G.); fabio.vanoglio@icsmaugeri.it (F.V.); raffaele.santoro@icsmaugeri.it (R.S.); 2 Istituti Clinici Scientifici Maugeri IRCCS, Scientific Direction of the Institute of Lumezzane, 25065 Lumezzane, Italy; 3 ASST Papa Giovanni XXIII, 24127 Bergamo, Italy; pfm.prometti@gmail.com

**Keywords:** balance, ADLs, diabetes, effectiveness, rehabilitation, recurrent falls, lower limb polyneuropathy

## Abstract

To date, little is known about the effects of motor rehabilitation in peripheral neuropathy (PN) patients with a history of recurrent falls (RFH). This study aimed to assess balance and the activities of daily living (ADLs) in elderly lower limb PN patients with and without RFH and to verify the effects of motor rehabilitation on balance and ADLs in these patients. We collected data from 64 lower limb PN patients, who underwent a conventional motor rehabilitation program: 35 patients had a history of recurrent falls, and 29 did not. The Berg Balance Scale (BBS) and motor FIM, before and after rehabilitation, were the outcome measures. After rehabilitation, lower limb PN patients with RFH had significantly higher scores in BBS and motor FIM (*p* < 0.001, for both) than at entry. The final BBS score and effectiveness in the BBS score of lower limb PN patients with RFH were lower than those of patients without RFH (*p* < 0.05 and *p* = 0.009, respectively). The study shows that conventional motor rehabilitation improves both balance and ADLs in patients, but balance improvement is lower in those with RFH. Thus, motor rehabilitation can be a therapeutic option for the management of these patients.

## 1. Introduction

Falls are common among people with peripheral neuropathy (PN), particularly in those who are older [1]. Peripheral neuropathies are neurologic disorders producing progressive damage to the peripheral nerve. They are generated by numerous causes, such as genetic and metabolic factors, autoimmune diseases, infections, drug or environmental toxicities, and malignancies. Diabetic neuropathy is the most common peripheral neuropathy (prevalence of 200–600 per 100,000) [2], whose pathogenesis is thought to be one of oxidative stress from disrupted metabolic pathways caused by hyperglycemia and dyslipidemia [3].

In PN patients, the progressive damage of the peripheral nerve produces motor and sensory deficits, which often result in balance impairment, mobility-related dysfunction, and alteration in gait features [4]. Disorders of balance and gait are particularly important in older PN patients, where they can compromise functional independence and contribute to the risk of falls and injury [5,6]. The incidence of falls in elderly patients with PN is up to 50% [1] and these patients are 23 times more likely to fall and 15 times more likely to report an injury compared with matched non-neuropathic subjects [7]. Falls, as a complication of PN, are increasingly being recognized as having a high major impact on the quality of life and overall health of older adults [8]. Moreover, falls can result in loss of confidence and reduced activity levels, leading to a loss of muscle strength and functional independence, change in bone microstructure, and fear of falling [8,9,10].

However, interest from researchers in the rehabilitation of PN patients with a history of recurrent falls (RFH) has been scarce [6]. Until now, the studies have focused on specific or conventional rehabilitation programs in PN patients without a history of falls or who are at risk of falls, showing that these patients have a significant improvement not only in balance but also in gait speed, disability, and health-related quality of life [5,8,11,12,13]. Therefore, to date, it is not known whether motor rehabilitation improves activities of daily living (ADLs) and balance in PN patients with RFH and whether RFH is associated with lower functional recovery in comparison to that of patients who do not fall. The clarification of these issues can give the physician tailored information to plan appropriate interventions to better manage PN patients.

The aim of this prospective cohort study was to assess balance and ADLs in lower limb PN patients with RFH with respect to patients without RFH and to verify the effects of motor rehabilitation on the same outcomes.

## 2. Materials and Methods

### 2.1. Patients

The study consisted of a secondary analysis of data from our database evaluating the impact of chronic diseases on balance and functional capacities in older patients admitted to our institute for rehabilitation between January 2016 and December 2018.

From this database, we retrospectively included old patients aged 66–90 years, admitted to inpatient rehabilitation because of disability related to lower limb PN associated with metabolic diseases [14] and with a history of recurrent falls. The diagnosis was based on clinical evaluation and electromyography (EMG) performed in our institute or another hospital of our health district before the patient’s admission to the study. We excluded patients who did not provide informed consent and those who had acute medical diseases, cancer, concomitant neurological pathologies, and orthopedic/surgical conditions causing, per se, a locomotor disability.

All patients admitted to the study provided their written informed consent to use their clinical data for scientific research. The study was conducted in accordance with the principles of the Declaration of Helsinki.

### 2.2. Assessments

During their in-hospital stay, all patients were examined by a qualified team of physiatrists and geriatricians, both before and after rehabilitation.

At admission, we recorded the patient’s demographic characteristics and comprehensive clinical data, including Body Mass Index (BMI), length of in-hospital stay (days), electromyography (EMG) findings, cognitive impairment (evaluated with the Mini-Mental State Examination [15]), presence of diabetes, comorbidities (assessed with the Cumulative Illness Rating Scale [16]), and whether they had a history of falls.

According to the presence or absence of recurrent falls, we identified two groups of people and defined patients with RFH as those who reported four or more falls in the previous year and patients without RFH as those without a history of falls. This selection was performed on the basis of previous studies [17].

Both before and after rehabilitation, we used scales of demonstrated validity and sensitivity to evaluate balance, ADLs, pain severity, passive range of motion (ROM), and muscle strength.

Balance was assessed with the Berg Balance Scale (BBS) [18,19]. This scale includes 14 items assessing capacity to maintain positions of varying difficulty and perform specific functional tasks. Each item is scored from 0 (unable to perform the task) to 4 (normal performance of a task) giving a total BBS score ranging from 0 to 56. Higher scores describe the best condition.

The patient’s degree of independence and need for assistance in performing ADLs were evaluated with the Functional Independence Measure (FIM) [20]. This scale comprehends 18 items (scored from 0 to 7); it can be subdivided into a 13-item motor sub-scale (motor FIM) and a 5-item cognitive sub-scale (cognitive FIM). In detail, 13 FIM motor sub-items were eating, grooming, bathing, dressing the upper body, dressing the lower body, toileting, bladder control, bowel control, transfer to bed/chair/wheelchair, transfer to toilet, transfer to tub/shower, walk or wheelchair, stairs), while 5 FIM cognitive sub-items were comprehension, expression, social interaction, problem-solving, memory. The motor sub-scale score ranges from 13 to 91, while the cognitive sub-scale score from 5 to 35. The maximum total FIM score is 126. Higher scores correspond to the best condition [20].

Pain intensity was assessed with an 11-point Visual Numeric Scale (VNS) scored from 0 (no pain) to 10 (intolerable pain) [21].

Muscle strength of hip flexor muscles and quadriceps was measured with the Muscle Strength Grading Scale (Oxford Scale) [22], scored from 0 (no movement) to 5 (muscle contracts against full resistance). The sum of the strength of the hip flexor muscles and quadriceps was considered in the current study.

Passive hip and knee ROM in flexion were evaluated using a manual goniometer [23], and the sum of the single ROM was considered.

### 2.3. Outcome Measures

We considered the total scores and items of BBS and motor FIM, before and after rehabilitation, and gain and effectiveness in BBS and motor FIM after rehabilitation as outcome measures.

Effectiveness represents the proportion of potential improvement obtained during rehabilitation, computed as (final score − initial score)/(maximum score − initial score) × 100. Therefore, effectiveness is 100% if a patient reaches the top score at the end of the rehabilitation program [24].

### 2.4. Rehabilitation Program

The rehabilitation program was carried out in an in-patient setting and was tailored to each patient’s clinical characteristics. It was discussed at admission and reevaluated bi-monthly by a multidisciplinary rehabilitation team, composed of physicians, nurses, physiotherapists, and an occupational therapist. The rehabilitation program included conventional motor rehabilitation, occupational therapy, nursing rehabilitation inward activities, electrostimulation of quadriceps muscles, and, in the presence of pain, transcutaneous electrical nerve stimulation, ultrasound or laser, at the physician’s discretion. Rehabilitation began the day after admission and was stopped when patients showed no further improvement through the rehabilitation training in the opinion of the rehabilitation team.

In detail, motor rehabilitation (330 min/week per 6 days/week) was performed for the entire duration of the in-hospital stay, while occupational therapy (150 min/week) was executed only in the last 2 weeks of the in-hospital stay. Motor rehabilitation was focused on exercises to improve muscle strength, joint mobility, balance, postural stability, changing the position of the body, walking, and the performance of ADLs. It consisted of lower limb strengthening and a passive range of motion exercises, balance exercises in sitting and standing positions (two legs stance, one-leg stance), supine-to-sit and sit-to-stand and vice versa exercises, gait training (walker, parallel bars, crutches, canes), and ADLs training (self-care skills, bathroom skills, climbing stairs).

### 2.5. Statistical Analysis

Statistical analyses were performed using the software Statistica Version 6. The assessment of the normality of the data was performed using the Shapiro–Wilk test and the descriptive statistic (mean ± SD or number) was used. Student’s *t*-test and χ2 tests (Fisher exact or Pearson, as appropriate) were performed to assess the differences within and among groups or sub-groups (i.e., diabetes). Pearson correlation coefficients were employed to examine the relationship between variables. In the study, we considered correlations ≥0.30 only, interpreting as moderate those with an r-value in the range of 0.30–0.50, and as strong with r > 0.51) [25]. *p* values < 0.05 were considered statistically significant.

## 3. Results

During the study period, 71 patients with disability related to lower limb PN associated with metabolic diseases were present in our database. Of these, 35 patients had a history of four or more falls, 7 had one fall, and 29 had any falls. We excluded from the statistical analyses patients with a history of one fall and, thus, we considered a total of 64 patients.

Table 1 reports the demographic and clinical characteristics of PN patients with and without RFH showing no statistical differences between the two groups.

### 3.1. Total Berg Balance Scale and Motor FIM

Table 2 describes the pre-to-post rehabilitation profiles of clinical parameters of PN patients with and without RFH.

**Figure 1:**
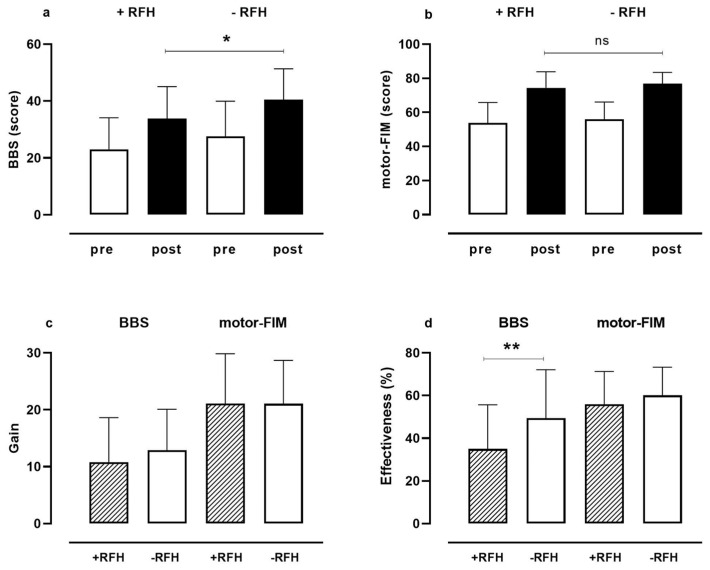
Motor FIM (**a**) and BBS (**b**) pre (white bar) to post (black bar) rehabilitation, and gain (**c**) and effectiveness (**d**) in the same outcome measures were shown in lower limb PN patients with (+) and without (−) history of recurrent falls (RFH). Legend: motor FIM: motor Functional Independence Measure; BBS: Berg Balance Scale. * refers to *p* < 0.05 for the difference in BBS between the two groups at the end of rehabilitation, and ** to *p* = 0.009 for difference in effectiveness in BBS score. ns referred to not significant.

Figure 1 reports BBS and motor FIM scores pre- and post-rehabilitation, gain, and effectiveness in PN patients with (+) and without (−) a history of recurrent falls (RFH).

Before rehabilitation, there were no differences in total BBS and motor FIM scores between the two groups of patients (Figure 1a,b—white bars). After rehabilitation (Figure 1a,b—black bars), all patients with and without RFH significantly improved their scores both in BBS and motor FIM (*p* < 0.001, for both) than at entry. However, as we showed in Figure 1, the final BBS score (panel a, *p* < 0.05) and effectiveness in BBS score (panel d, *p* = 0.009) of patients with RFH were significantly lower than those without RFH, suggesting a lower balance improvement in patients previously conditioned by repeated falls. On the contrary, there were no differences in motor ability (Figure 1b, *p* = 0.233) and effectiveness in motor FIM (Figure 1d, *p* = 0.252) between patients with or without a history of recurrent falls.

Concerning specific patients’ comorbidities (e.g., diabetes), we performed a further analysis in a sub-group of PN patients with RFH and diabetes with respect to PN patients without RFH and diabetes. Table 3 describes pre-to-post rehabilitation profiles of clinical parameters showing that PN patients with RFH and diabetes had, in comparison to PN patients without RFH and diabetes, lower BBS scores before rehabilitation (*p* = 0.031) and lower BBS (*p* = 0.011), motor FIM (*p* = 0.031) and effectiveness in BBS score (*p* = 0.024) after rehabilitation.

Table 4 and Table 5 report significant correlations between BBS and motor FIM outcomes and the clinical and demographic characteristics of PN patients at admission, as assessed by Pearson’s correlation.

Effectiveness in BBS and motor FIM were moderately related to age (r = −0.33 and r = −0.36, respectively, for both, *p* < 0.001), cognitive FIM at admission (r = 0.32, *p* < 0.05, and r = 0.35, *p* < 0.001; respectively, for BBS and motor FIM) and cognitive-FIM at discharge (r = 0.36 and r = 0.41, respectively, for both *p* < 0.001).

### 3.2. Single Items of Berg Balance Scale

Before rehabilitation, patients with RFH had lower BBS scores in transfers (*p* = 0.039), reaching forward with an outstretched arm (*p* = 0.024), and retrieving objects from the floor (*p* = 0.049) in comparison to patients without RFH.

After rehabilitation, the scores of all BBS items were higher than those of admission in both groups (*p* < 0.001, for all) with the exception of sitting unsupported (*p* = ns, in both groups).

At the same time, patients with RFH showed, in comparison to patients without RFH, lower scores in standing unsupported (*p* = 0.023), standing to sit (*p* = 0.043), retrieving objects from the floor (*p* = 0.015), placing an alternate foot on the stool (*p* = 0.021), and standing with one foot in front (*p* = 0.014), and lower gain in standing to sitting (*p* = 0.035) and standing on one foot in front (*p* = 0.005).

### 3.3. Single Items of Motor FIM

Before rehabilitation, PN patients with RFH did not differ from patients without RFH in all motor FIM items. At the end of rehabilitation, the scores of all motor FIM items were higher than admission in both groups (*p* < 0.01 for all). After rehabilitation, in comparison to patients without RFH, PN patients with RFH had a lower score only in transfer to tub/shower (*p* = 0.014).

## 4. Discussion

This study aimed at evaluating the effects of a conventional motor rehabilitation program in patients with disability related to lower limb PN with a history of recurrent falls. The employment of motor FIM and BBS scales before and after rehabilitation showed that, at the end of rehabilitation, all lower limb PN patients improved their performances with respect to those observed at admission. However, PN patients with RFH had lower total scores and effectiveness in BBS compared to patients without RFH suggesting a lower balance improvement. In the literature, there are several studies on the rehabilitation of PN patients [5,7,11,12,13], but none of these studies analyzed PN patients with RFH undergoing rehabilitation; for this reason, it is not possible to compare our findings with those from other authors.

In the study, we used a conventional rehabilitation program that included exercises for balance and ADLs and was the same for both patient groups. Furthermore, before rehabilitation, PN patients with RFH did not differ from those without RFH in age, pain, ROM, lower limb muscle strength, and cognitive FIM, but at the end of rehabilitation, they had a poorer improvement in balance. This finding suggests that in our sample the lowest improvement of balance in PN patients with RFH was not due to the rehabilitation program, age, pain, and cognitive functions, but to their more severe deficits in proprioceptive and vestibular function, postural stability, muscular strength [5,10,26,27] and also to their poorer balance reserves [28,29,30]. These latter were adequate to ensure postural stability in basal conditions but were not adequate to produce an improvement in balance similar to that observed in patients without RFH during rehabilitation.

At the end of the rehabilitation, PN patients with RFH showed a lower gain in standing to sitting and standing on one foot in front. These BBS items are highly unstable tasks and, between them, standing on one foot in previous studies is considered to be the most challenging activity for PN patients [31,32]. Standing to sitting and standing on one foot in front require higher levels of balance, muscle strength, and cognitive function [28,29], and their poor improvement may have contributed to lower scores and effectiveness in balance in PN patients with RFH.

Unlike the balance, the improvement of ADLs in PN patients with RFH did not differ from that of patients without RFH. Balance is crucial for the performance of normal physical activities and is one of the prerequisites for executing ADLs and transfers without difficulty [33,34].

However, in our study, there were no differences in ADLs between the two groups, although PN patients with RFH had lower levels of balance at the end of rehabilitation than those of patients without RFH.

The presence of diabetes can make a difference. Diabetic patients with a history of falls had lower levels of balance and cognitive function than those patients without diabetes and a history of falls before rehabilitation. They continued to have lower balance levels and cognitive function at the end of rehabilitation but at this time they also had lower ADL levels. This indicates that diabetic patients with RFH had a course of disease different from diabetic patients without RFH. This may be explained by the fact that they showed at admission a poorer cognitive function, which has been demonstrated to be a complication of diabetes and a factor influencing the standing balance and postural sway of these patients [35]. In addition, some authors reported that diabetic patients have slower reaction times, decreased sensitivity of proprioceptive and vestibular function, and greater postural instability in comparison to patients without diabetes [36,37].

The Pearson’s correlation showed relationships between the percentage of improvement in BBS and motor FIM and age and cognitive function. In particular, the percentage of improvement in BBS and motor FIM was lower in older PN patients and PN patients with cognitive function impairment. Age and cognitive functions are factors that cannot be changed with the rehabilitation program. Thus, the associations found in the study suggest that longer and more intensive rehabilitation programs are needed to increase the effectiveness of balance and ADLs in older PN patients and in those with cognitive impairment [35,38].

The study also evaluated single items of BBS and motor FIM in PN patients before and after rehabilitation. Before rehabilitation, PN patients with RFH had lower scores in transfers, reaching forward with an outstretched arm and fetching objects from the floor. Conversely, at the end of rehabilitation, they differed from patients without RFH in the gain of standing to sitting and standing on one foot in front. These data can help health staff to better manage these patients.

On the one hand, they indicate that not only transfers, as already demonstrated by other authors [39,40], but also reaching forward with an outstretched arm and fetching objects from the floor may be activities with a fall risk for patients with RFH. Thus, these patients have to be educated to perform these tasks correctly and safely [28].

On the other, they suggest that standing to sitting and standing on one foot in front are tasks that in PN patients with RFH have poorer improvement with rehabilitation and, thus, need more intensive and specific rehabilitation exercises.

### Limitations

Despite these considerations, the current study has some limitations.

The patient sample was not representative of the general population, because only PN patients requiring hospital rehabilitation were admitted to the study. The study analyzed only some clinical and demographic variables that may have influenced the functional status and balance and did not consider variables such as duration of illness and sensory system deficits.

The current study employed a conventional motor rehabilitation program; therefore, the findings may be different from other rehabilitation programs. The study considered patients with repeated falls and excluded from the analysis patients with a history of one fall because only one fall in elderly patients can be linked to a lack of balance but also to other causes non-PN-related [41,42].

## 5. Conclusions

This study shows that in PN patients with RFH conventional motor rehabilitation improves both balance and ADLs. However, balance improvement is lower in patients previously conditioned by repeated falls suggesting that motor rehabilitation can be a therapeutic option for the management of these patients, in particular focusing on balance recovery. At the same time, the evaluation of single balance tasks and single ADLs in PN with RFH allows the identification of the tasks and activities that are at higher risk for falling and need more intensive rehabilitation.

## Figures and Tables

**Table 1:** Demographic and clinical characteristics of peripheral neuropathy patients with and without a history of recurrent falls.

	PN with Recurrent Falls (n = 35)	PN without Recurrent Falls (n = 29)	*p*-Value
Age, years	83.22 ± 4.29	81.06 ± 5.63	0.072
Male/Female, n	11/24	13/16	0.485
Diabetes mellitus: Yes/No	14/21	15/14	0.540
CIRS severity score	2.56 ± 0.32	3.05 ± 3.57	0.419
Body Mass Index, Kg/m^2^	28.61 ± 6.70	27.52 ± 6.21	0.504
EMG: demyelinating neuropathy, axonal neuropathies (sensitive and motor ones), axonal demyelinating neuropathy	4/20/11	2/20/7	0.730
MMSE *	21.70 ± 4.81	22.17 ± 5.44	0.772

Legend: Data are expressed as mean and SD or absolute numbers. PN = peripheral neuropathy; CIRS = Cumulative Illness Rating Scale Geriatrics; EMG = electromyography; MMSE = Mini-Mental State Examination. * Data were available in 41 patients, of which 24 with recurrent falls and 17 without. Comparison between groups was performed by Student’s *t*-test and chi-square. A *p* < 0.05 was considered statistically significant.

**Table 2:** Profiles of clinical parameters in PN patients with (n = 35) and without a history of recurrent falls (n = 29).

	PN with Recurrent Falls (n = 35)	PN without Recurrent Falls (n = 29)	*p*-Value
**At admission**			
VNS pain, score	6.42 ± 1.83	6.41 ± 2.48	0.978
ROM, °	455.00 ± 46.57	456.51 ± 51.15	0.901
Muscle strength, score	12.77 ± 1.84	13.15 ± 1.49	0.371
Cognitive-FIM, score	26.31 ± 3.67	27.37 ± 3.44	0.239
**At discharge**			
VNS pain, score	3.71 ± 1.60	3.31 ± 1.77	0.342
ROM, °	455.71 ± 77.45	477.58 ± 29.29	0.155
Muscle strength, score	14.71 ± 1.63	14.86 ± 1.15	0.684
Cognitive-FIM, score	26.77 ± 3.87	28.10 ± 3.72	0.168
Length of hospital stay, days	35.37 ± 12.64	34.96 ± 9.79	0.888

Legend: Data are expressed as mean and SD. Abbreviations: PN = peripheral neuropathy; FIM = Functional Independence Measure; VNS = Visual Numeric Scale; ROM = range of motion. Comparison between groups was performed by Student’s *t*-test. A *p* < 0.05 was considered statistically significant.

**Table 3:** Profiles of demographic and clinical characteristics and outcomes measures of diabetic patients with a history of recurrent falls and non-diabetic patients without a history of recurrent falls.

	Diabetes and Falls (n = 14)	No Diabetes and No Falls (n = 14)	*p*-Value
**At admission**			
Age, years	81.57 ± 5.10	80.28 ± 5.29	0.519
Male/Female, n	8/6	6/8	0.449
Body Mass Index, Kg/m^2^	29.74 ± 8.58	25.66 ± 5.01	0.136
CIRS, score	2.59 ± 0.28	3.66 ± 5.14	0.444
MMSE *, score	20.17 ± 4.69	20.71 ± 6.13	0.826
VNS pain, score	6.00 ± 2.11	6.78 ± 2.39	0.365
ROM, °	455.71 ± 39.46	448.50 ± 65.17	0.725
Muscle strength, score	12.28 ± 1.54	13.32 ± 1.74	0.108
Cognitive-FIM, score	25.21 ± 3.26	28.42 ± 2.82	0.009
Berg Balance Scale, score	19.71 ± 11.03	29.64 ± 12.04	0.031
Motor-FIM, score	49.21 ± 13.82	56.85 ± 10.53	0.111
**At discharge**			
VNS pain, score	3.78 ± 1.96	3.50 ± 1.78	0.690
ROM, °	470.71 ± 24.09	483.57 ± 25.82	0.162
Muscle strength, score	14.14 ± 1.87	15.07 ± 1.26	0.136
Cognitive-FIM, score	25.07 ± 3.17	29.21 ± 2.75	0.001
Berg Balance Scale, score	31.00 ± 12.47	42.57 ± 9.97	0.011
Effectiveness in Berg Balance Scale, %	32.70 ± 22.80	52.89 ± 21.80	0.024
Gain in Berg Balance Scale, score	11.28 ± 9.10	12.92 ± 7.62	0.608
Motor-FIM, score	69.78 ± 12.96	78.14 ± 4.52	0.031
Effectiveness in motor-FIM, %	50.12 ± 21.27	60.78 ± 11.71	0.112
Gain in motor-FIM, score	20.42 ± 10.74	22.07 ± 9.19	0.667

Legend: Data are expressed as mean ± SD or absolute number. CIRS = Cumulative Illness Rating Scale Geriatrics; VNS = Visual Numeric Scale; MMSE = Mini-Mental State Examination; ROM = range of motion; FIM = Functional Independence Measure. Comparison between groups was performed by Student’s *t*-test. A *p* < 0.05 was considered statistically significant. * For MMSE, data were available from 12 diabetic patients with a history of falls and 8 non-diabetic patients without a history of falls.

**Table 4:** Moderate and significant relationships between Berg Balance Scale outcomes and clinical and demographic characteristics of PN patients (n = 64).

		BBS	
	Score at Admission	Score at Discharge	Effectiveness
Age, years	−0.23	−0.28 *	−0.33 **
Cognitive-FIM score at admission	0.25 *	0.31 *	0.32 *
Cognitive-FIM score at discharge	0.24	0.35 **	0.36 **

Legend: FIM = Functional Independence Measure. Correlation between variables was performed by Pearson’s correlation coefficient (r); * *p* < 0.05; ** *p* < 0.001.

**Table 5:** Moderate and significant relationships between motor-FIM outcomes and clinical and demographic characteristics of PN patients (n = 64).

		Motor FIM	
	Score at Admission	Score at Discharge	Effectiveness
Age, years	−0.13	−0.29 *	−0.36 **
Muscle strength, score at admission	0.46 **	0.23	−0.08
Muscle strength, score at discharge	0.32 *	0.23	0.04
Cognitive-FIM score at admission	0.06	0.30 *	0.35 **
Cognitive-FIM score at discharge	0.09	0.35 **	0.41 **

Legend: FIM = Functional Independence Measure. Correlation between variables was performed by Pearson’s correlation coefficient (r); * *p* < 0.05; ** *p* < 0.001.

## Data Availability

Data supporting reported results can be required from the corresponding author.
